# Optimized design and performance evaluation of a highly precise variable rate mis-planting and replanting potato electronic-metering mechanism

**DOI:** 10.3389/fpls.2025.1531377

**Published:** 2025-05-16

**Authors:** Abdallah Elshawadfy Elwakeel, Ahmed Elbeltagi, Ali Salem, Ahmed Z. Dewidar

**Affiliations:** ^1^ Agricultural Engineering Department, Faculty of Agriculture and Natural Resources, Aswan University, Aswan, Egypt; ^2^ Agricultural Engineering Department, Faculty of Agriculture, Mansoura University, Mansoura, Egypt; ^3^ Structural Diagnostics and Analysis Research Group, Faculty of Engineering and Information Technology, University of Pécs, Pécs, Hungary; ^4^ Civil Engineering Department, Faculty of Engineering, Minia University, Minia, Egypt; ^5^ Prince Sultan Bin Abdulaziz International Prize for Water Chair, Prince Sultan Institute for Environmental, Water and Desert Research, King Saud University, Riyadh, Saudi Arabia; ^6^ Department of Agricultural Engineering, College of Food and Agriculture Sciences, King Saud University, Riyadh, Saudi Arabia

**Keywords:** precision agriculture, internet of things, machine learning, qualified index, mis-planting index, seed monitoring system

## Abstract

**Introduction:**

Precise seed placement during potato planting critically determines crop distribution and density, yet mis-planting remains a persistent agricultural challenge. Current manual detection and correction methods introduce inefficiencies, increase labor costs, and risk human error.

**Methodology:**

To address these limitations, this study developed and evaluated a high-precision variable-rate electronic metering mechanism (EMM) capable of automated mis-planting detection and replanting under controlled laboratory conditions. The EMM was built to operate at different planting distances and travel speeds, with its design focusing on finding the best mechanical setup before testing it in the field at four different planting distances (24.12, 31.06, 34.87, and 41.24 cm) and five speeds (2.13-6.11 km/h).

**Results:**

The obtained results demonstrated optimal stability at lower speeds (2.13-3.07 km/h), where sensor accuracy remained consistent, achieving peak performance (QI=98.7%, RI=100%, minimal MPI) at 41.24 cm spacing and 2.13 km/h. Performance degraded significantly at higher speeds (3.94-6.11 km/h), with factorial analysis confirming both speed and spacing as statistically significant factors affecting all indices. Furthermore, the total cost of the developed system was approximately $130 USD.

**Discussion:**

Future experiments will include further field experiments to study the influence of field variables such as soil type, surface irregularity, and environmental disturbances on the performance of the EMM.

## 1 Introduction

In 2021, potatoes were the sixth most produced crop in Egypt, with an annual yield of 6.9 million tons (Statista, 2024). The potato planter is an indispensable instrument for the advancement of the potato industry, as its operational efficiency has a direct impact on the growth and development of potatoes, which consequently influences overall yields (Zhang et al., 2024). In recent years, significant strides have been made in the mechanization of potato planting; however, the degree of automation and intelligence remains relatively limited (Liu et al., 2021; Li et al., 2022a). The quality of planting is a critical factor affecting yield levels. In situations of land limitation, it is imperative to improve yields through technological innovations to ensure a reliable product supply, in conjunction with technical strategies like cultivar selection, culture conditions, and planting methodologies (Elsayed et al., 2023; Malkani et al., 2024; Taha et al., 2024; Elwakeel et al., 2025; Farag Taha et al., 2025). Uniformity in planting is an essential indicator of planting quality. Reduced intraspecific competition, increased yield, equal root and plant development, and optimum growth area for each plant are all benefits of evenly dispersed seeds (Zhan et al., 2010). A critical factor affecting precision sowing/planting is the lack of seeds. As a result, several researchers have sought to address the problem of mis-seeding or mis-planting (Zhang et al., 2022; Yang et al., 2024). Precise management of various critical indicators is essential in the potato planting process. The occurrence of mis-planting and duplicated plantings, resulting from fluctuations in the number of seed potatoes in the seed spoon, considerably impacts planting quality (Qiu et al., 2023b; Zhang et al., 2024, 2024). The current sowing/planting machine suffers from leakage attributable to unavoidable circumstances, such as the obstruction of the seed row disc opener and seed guide tube, slippage of the ground wheel, and other inevitable causes (Jafari et al., 2010; Cay et al., 2017, 2018; He et al., 2017, 2019a, 2021; Patrício and Rieder, 2018; Karimi et al., 2019; Liu et al., 2019a, b; Jing et al., 2020; Xie et al., 2021; Zhang et al., 2022).

Seed leakage remains a critical challenge in agricultural planting operations, primarily stemming from two factors: seeder design limitations and variable field conditions (Zhu et al., 2015). These persistent issues frequently result in mis-seeding events that are particularly difficult to prevent using conventional methods. To address this problem, advanced monitoring systems coupled with automatic seed compensation mechanisms have emerged as an effective solution. By continuously tracking seed discharge and automatically replacing missed seeds, this technology significantly reduces leakage rates while simultaneously improving planter efficiency. This approach offers multiple benefits: reduced operational costs by minimizing manual replanting labor, prevention of yield losses from missed planting locations, and overall improved seeding accuracy. As demonstrated by (Zhang et al., 2022), automated monitoring and compensation systems represent the most direct and effective method for mitigating seeding-related yield losses, making their implementation essential for modern precision agriculture.

The seed discharge effect of the seeder is a critical factor influencing the overall quality of the planting operation, as it determines seed placement accuracy and uniformity. To enable real-time monitoring of the planter’s performance, early researchers developed a sensing mechanism by attaching high-precision sensors to the seed discharge tube beneath the seed dispenser, which generates electrical signals corresponding to seed flow dynamics. By analyzing the resulting signal variations—such as frequency, amplitude, and timing—operators can detect anomalies like missed seeds, double seeding, or irregular dispensing, thereby identifying improper sowing conditions (Fu et al., 2018; Li et al., 2021). This method provides an efficient, non-intrusive solution for optimizing seeding precision and improving crop yield potential. Where sensors have played a pivotal role in the evolution of mis-planting detection. And many researchers used many types of sensors such as, (Zhang et al., 2024) developed a potato planting system that employs the YOLOv7-tiny model to identify instances of missed and repeated planting. The results indicate that the detection accuracy for missed and repeated plantings reached 96.07% and 93.98%, respectively. (Liu et al., 2019b) created a device for detecting mis-seeds and reseeding in maize, employing a fiber optic sensor and an STM32F407 microcontroller. The results demonstrate that the accuracy of detecting mis-seeding exceeds 96%, the replanting rate exceeds 90%, and the replanting pass rate exceeds 95%. The sowing quality rate surpasses 90% when the tractor operates at a speed of 3 to 8 km/h. (Liu et al., 2019b) developed a sensor for quantifying the sowing of minute seeds. The photoelectric sensor and fiber sensor were utilized to calibrate the developed sensor for the detection process. The results demonstrate that the average monitoring accuracy of the developed sensor was 97.09%. and (Tang et al., 2022) designed a monitoring system for precision corn seed metering equipment with a long-belt finger-clip mechanism. The findings revealed that, at a peak velocity of 65 r/min, the average qualifying index increased to 80.225%, whilst the average reseeding index decreased to 11.075%, and the average mis-seeding index diminished to 8.7%.

Variable-rate planting systems (VRPS) have revolutionized modern agriculture by dynamically adjusting seeding rates in response to field conditions, optimizing resource efficiency, reducing input costs, and maximizing crop yields. However, despite their global success, VRPS adoption in Egypt remains limited due to the absence of locally developed systems and the high cost of imported solutions, which are financially out of reach for most smallholder farmers. To address this gap, researchers worldwide have made significant advancements in high-precision VRPS technologies. For instance, (Wang et al., 2022) designed an electronic metering mechanism (EMM) for maize planters using a fuzzy PID algorithm, achieving an impressive efficacy rate exceeding 95.81% while maintaining a reseeding rate below 10.11%. Similarly, (He et al., 2019b) developed an EMM for maize planters that achieved a remarkable seeding accuracy of 99.68% with a seeding coefficient of 0.85%. Earlier innovations include (Jafari et al., 2010), who engineered a DC motor-driven EMM for grain drills, while (He, 2013; Zhai JianBo et al., 2014) created stepper-motor-based EMMs for wheat and soybean planters. Additionally, (Li et al., 2015) contributed to the field by developing a hybrid DC and stepper motor EMM for precision corn planting, demonstrating the versatility and adaptability of these systems across different crops and machinery. These advancements highlight the potential for cost-effective, locally adaptable VRPS solutions that could benefit emerging agricultural markets like Egypt.

The identification of missing potato seeds and their subsequent replanting is now performed manually, leading to increased cultivation expenses and a greater probability of human error. The efficacy of the cultivation process and the density of plants per unit area might be significantly affected, as undetected absent potato seeds only become evident after germination, leading to losses for farmers. Previous research demonstrates that there is currently no effective technique for detecting missing potato seeds and transplanting them, apart from a study by Zhang et al. (2024), which utilized machine learning technology. This method is expensive, difficult to implement, and inefficient in open settings, especially those exposed to dust and other environmental factors. This study aims to utilize the Internet of Things (IoT) to develop and evaluate a highly precision variable rate EMM for potato planters, capable of detecting and replanting missing potato seeds.

## 2 Materials and methods

### 2.1 Main structure

**Figure 1:**
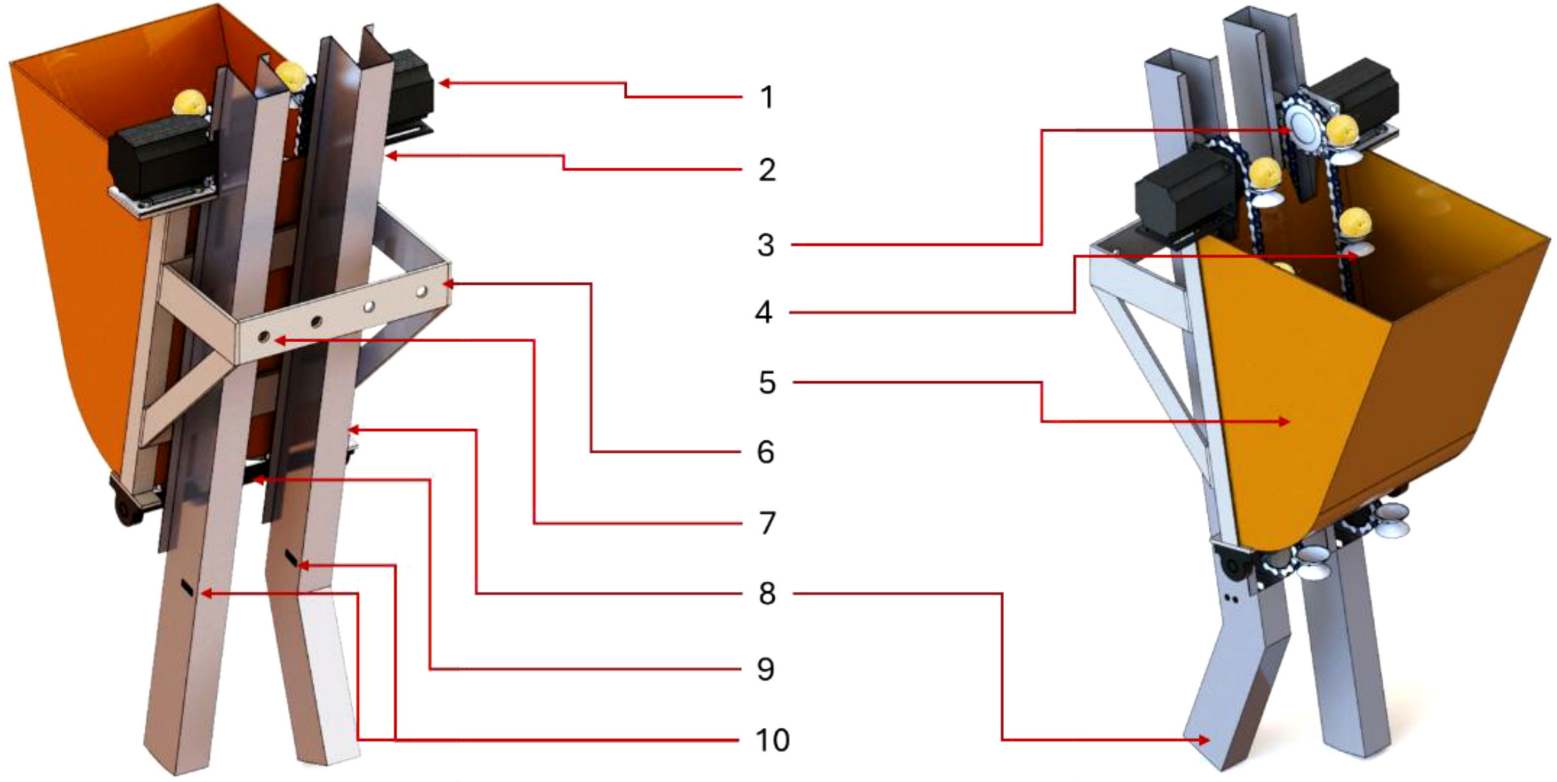
Main structure and key components of the developed EMM. Whereas (1). Stepper motor for planting unit, (2). Stepper motor for replanting unit, (3). Sprocket, (4). Double layer seed picking spoon (DLSPS), (5). Potato seeds hopper, (6). Main frame, (7). Fixing holes with test bench, (8). Normal potato seeds guide tube (NPSGT), (9). Compensatory potato seeds guide tube (CPSGT), (10). Ultrasonic sensors holes.

The detection and replanting of missing potato seeds during cultivation present a major agricultural challenge, as current practices rely heavily on manual labor to identify and correct mis-seeds—a method that is not only inefficient and costly but also susceptible to human error. To address this issue, this study introduces an innovative Electronic Metering Mechanism (EMM) designed to autonomously detect and replant missing seeds, eliminating the need for human intervention. The system integrates five key components: (1) a precision planting unit for initial seed placement, (2) an automated replanting unit to correct missed seeds, (3) a monitoring unit for the planting process (MUPP) to track seed distribution in real time, (4) a mis-seed detection unit (MSDU) equipped with advanced sensors to identify gaps in planting, and (5) a robust machine frame ensuring structural stability and operational efficiency. As illustrated in **Figure 1**, the EMM’s optimized design streamlines the planting process, enhancing accuracy while reducing labor dependency and operational costs—a transformative solution for modern potato farming.

**Figure 2:**
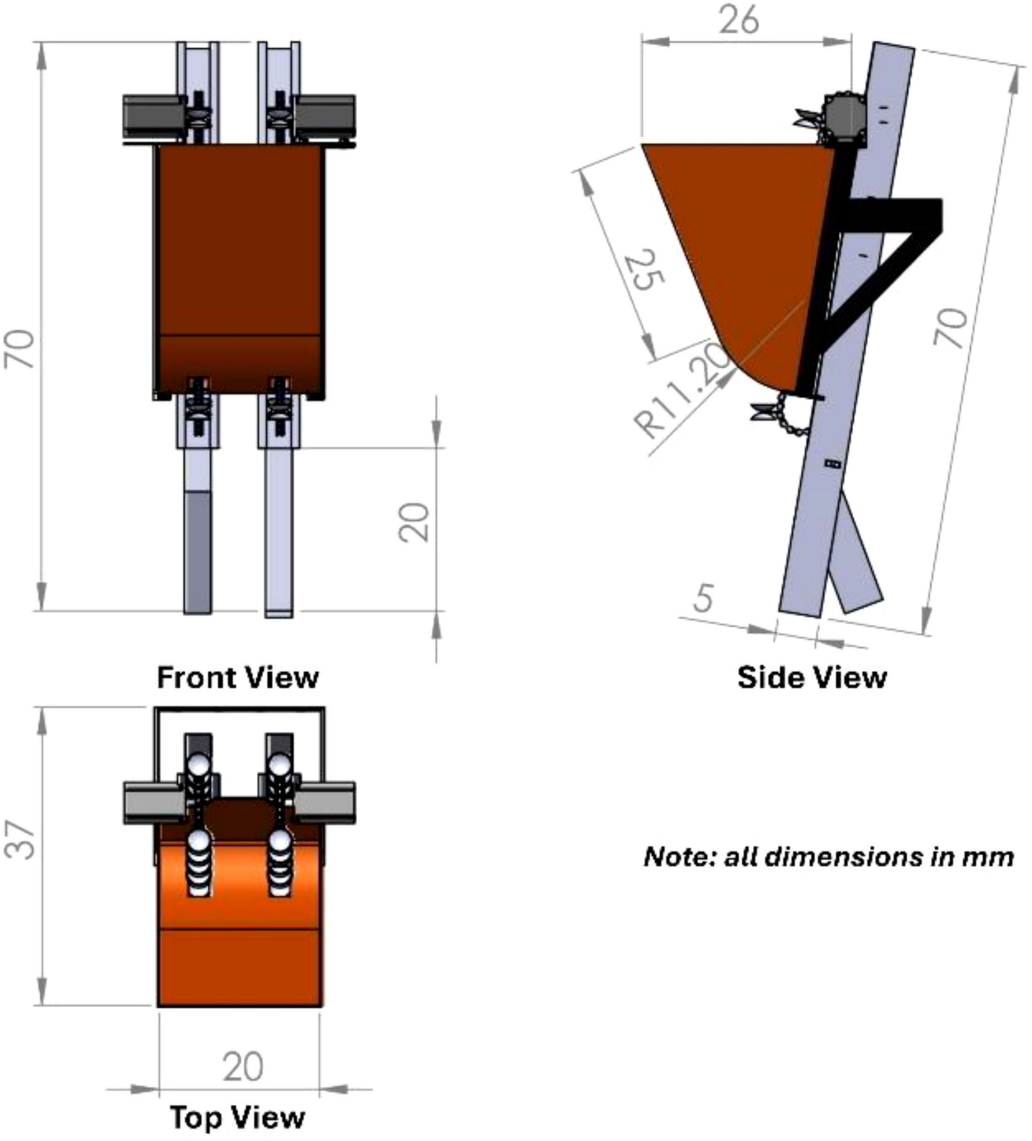
Detailed drawing of the developed EMM showing the main dimensions.

**Figure 2** illustrates that the overall height and width of the developed EMM are 70 cm and 37 cm, respectively. The NPSGT is inclined backward at 30°, but the CPSGT is linear and inclined forward at the identical angle. The EMM is equipped with a front-mounted potato hopper with an approximate capacity of 10 liters. This container is made of 1 mm thick galvanized sheet metal. The front and rear sides of the potato hopper are sloped at approximately 75° to facilitate the seamless passage of potato seeds during operation; furthermore, it is constructed without sharp edges to prevent the accumulation of seeds on one side.

**Figure 3:**
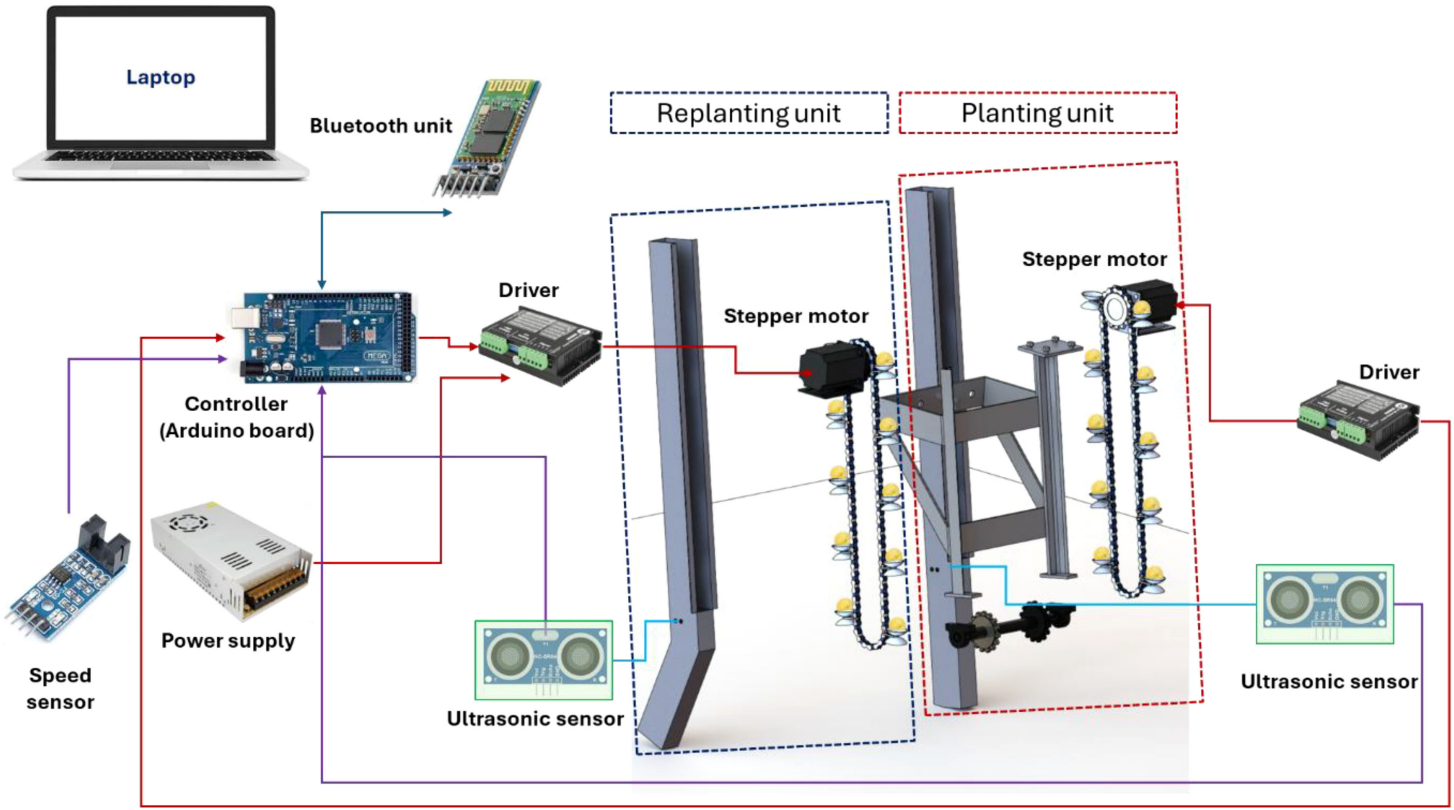
Connections, signal flowrate and operating map of both planting and replanting units.

**Figure 4:**
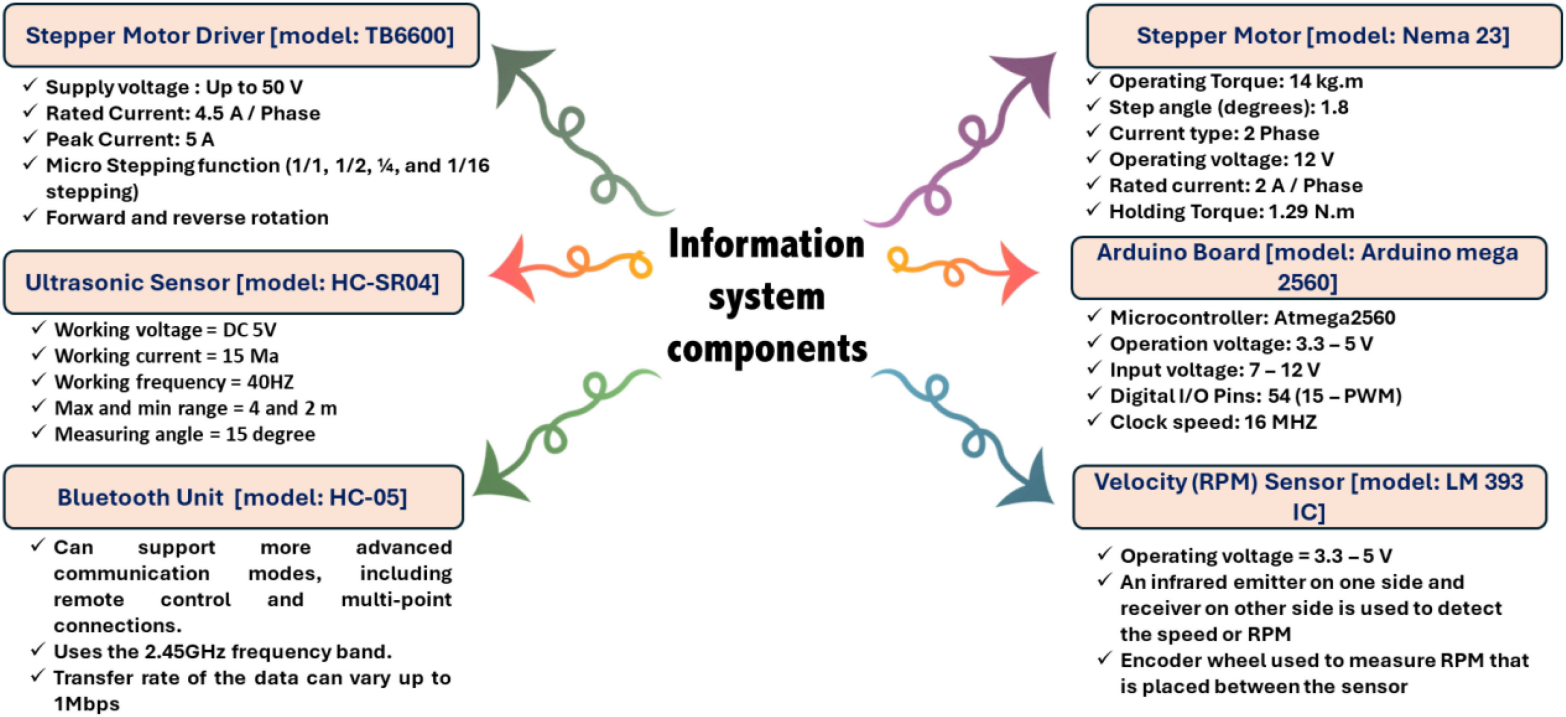
Specifications of the main electronic components of the developed EMM.

**Figure 3** illustrates the interconnections, signal flow rate, and operating schematic of both planting and replanting units. Where the planting and replanting units consist of many components, which are: (1) a stepper motor (model: Nema 23), (2) a stepper motor driver (model: TB 6600), (3) a ten DLSPS with a diameter of 4mm and a depth of 1cm, affixed to an each chain with a 10cm spacing between each, and (4) a sprocket with a diameter of 10cm. An Arduino board (model: Mega 2500 R_3_), a Bluetooth module (model: HC-05), and an infrared light speed sensor (model: LM 393 IC). Additionally, other auxiliary components were employed, including a laptop, breadboards, wires, a USB cable, and a converter (5–12 V). **Figure 4** illustrates the specifications of the main electronic components.

### 2.2 Analysis of the operating mechanism

**Figure 5:**
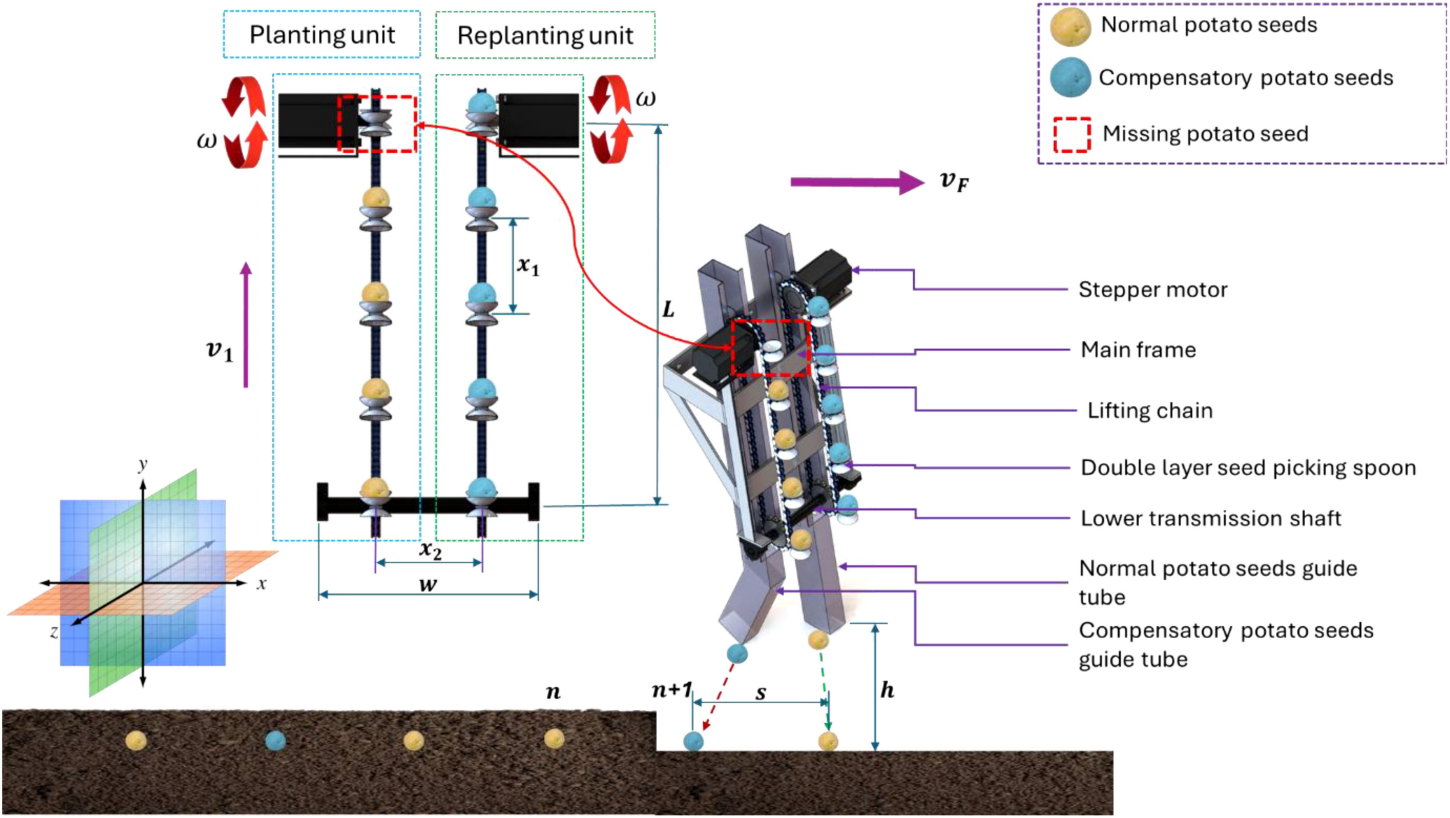
Schematic drawing of metering mechanism of both planting and replanting units.

The existing EMM now has an improved distribution mechanism. This process efficiently decreases the drop height of the potato seed. It mitigates the incidence of rebound effects between the seed and the soil during high-velocity precision seeding. The precision, consistency, and alignment of horizontal and vertical seed planting have improved. The component that powers the revolving sprocket during the planting procedure is the stepper motor. The DLSPS concurrently rotates the individual potato seed in a counterclockwise direction, as depicted in **Figure 5**. Thereafter, the DLSPS transports the individual potato seed to the potato seed guide tube with ease. The initial stage of the migration process has been finalized. The potato seed is placed on the guide chain, which rotates concurrently with the EMM as it traverses the guide tube intended for potato seeds. The combined effects of gravity and support enable the seamless descent of the potato seed to the specified delivery location. Planting the potato seed at a superficial depth in the soil impedes its growth and obstructs its development. The second migration operation has been verified as having occurred. **Figure 5** illustrates how secondary distribution reduces the speed at which potato seeds fall into the seed ditch, enabling efficient and smooth seed transportation and delivery.

**Figure 6:**
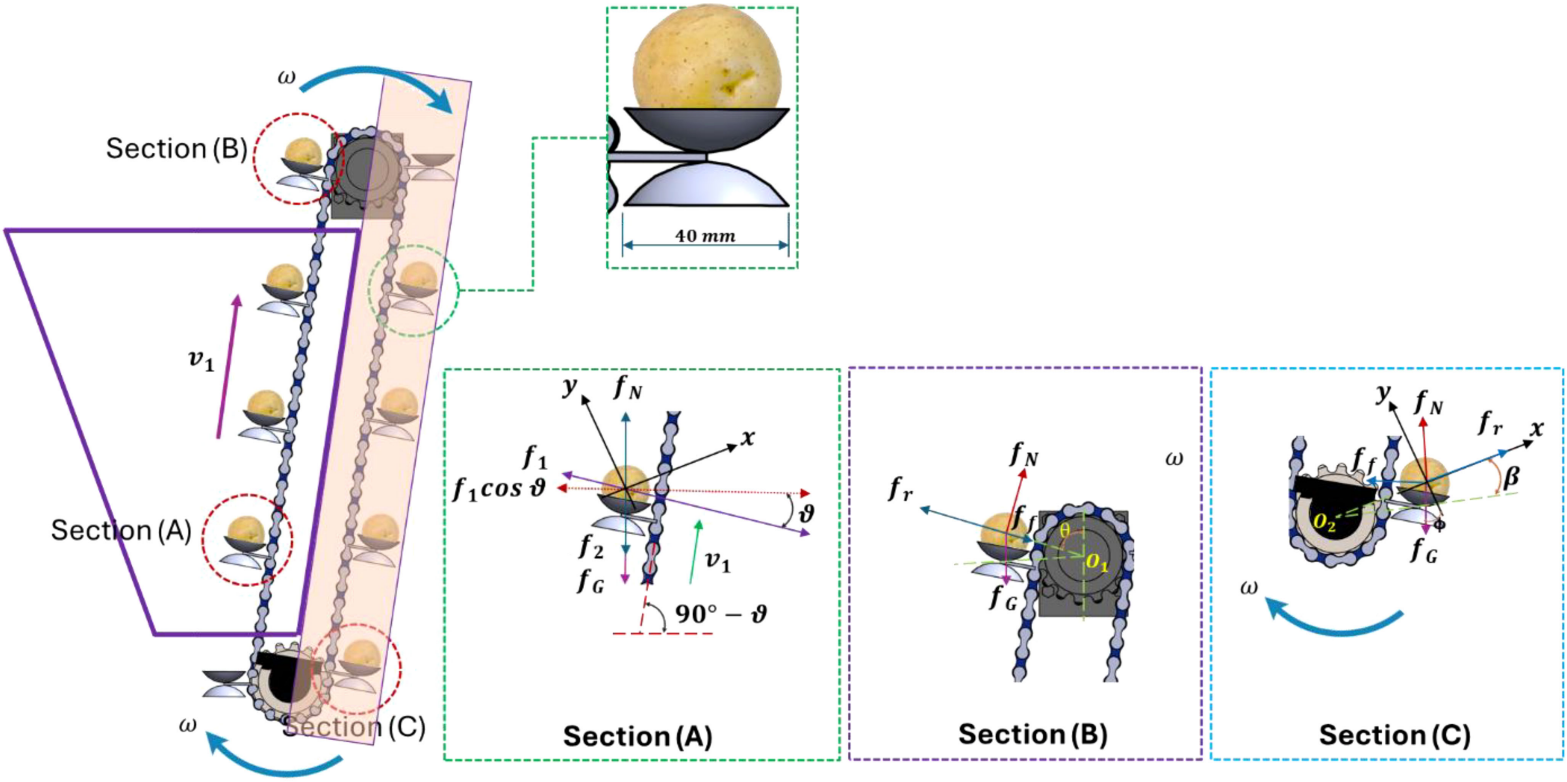
Force analysis diagram of smooth transport and delivery of potato seed during the developed EMM.


The researchers performed a mechanical investigation of potato seed movement to evaluate their stability during transportation and delivery. Moreover, they investigated the circumstances that allow the seeds and the seed guide blade to sustain relative balance (Wang et al., 2017). A spatial Cartesian coordinate system with axes X, Y, and Z is formed by identifying the rotational center of the EMM shaft as the origin (O). This procedure is illustrated in **Figure 6**. Upon the insertion of a single potato seed into the seed guide tube, it turns counterclockwise in tandem with the seed guide chain. Following this spin, the force applied by the seed is measured and analyzed. It is essential to prevent the seed from being expelled from the feeding fork to sustain a favorable relationship between the seed and the feeding fork. The force applied to the potato seed in the slip direction concerning the feeding fork is absorbed within the rotating plane (xoy) of the seed guide chain (Equation 1). **Figure 6** (*section A*) depicts a complex network of stresses exerted on the potato seeds, corresponding to connection (Qiu et al., 2023a; Elwakeel et al., 2024c; Yang et al., 2024):

(1) $$\left\{ \begin{array}{c} f_{N}=f_{2}+f_{G}\\ f_{f}=f_{1} \end{array}\right.$$

where, *f*_2_ is the is the longitudinal synergistic pressure between seed potatoes, N, *f_G_* is the gravitational force of potato seed, N, *f_f_* is the friction force between potato seed and the DLSPS, N, *f*_1_ is the transverse synergistic pressure between seed potatoes, N.

Upon reaching the driven sprocket, the seed potato will be thrust to the back of the preceding seed harvesting spoon. **Figure 6** (*section B*) depicts the force diagram, and the necessary condition for the seed potato to maintain relative stability and prevent ejection must conform to the following relationship (Equation 2):


(2) {fN=fGsinθ ff+fGsinθ≥fr

where, *f_r_* is the inertia force, N; *θ* is the rotation angle of the DLSPS, ˚.

In the potato seed ejection phase, heightened velocity generates greater inertial force; a diminished rotational angle of the DLSPS leads to the ejection of the seed potato, causing it to diverge from the DLSPS trajectory. At the initial seed drop position, with the seed potato’s center of mass as the origin, build a Cartesian coordinate system where the x-axis coincides with the direction of the inertial force and the y-axis is perpendicular to the inertial force, as depicted in **Figure 6** (*section C*). The forces exerted on the seed potato conform to the subsequent relationship:


(3) $$\left\{ \begin{array}{l} f_{N}\cos\beta +f_{f}sin\beta -f_{G}cos\varphi =0\\ f_{N}sin\beta +f_{f}cos\beta -f_{G}sin\varphi \leq f_{r} \end{array}\right.$$

where, *β* is the residual angle of the angle between the inertia force $f_{r}$ and the support force $f_{N}$ , (˚); φ is the angle between gravity $f_{G}$ and the y-axis.

The relationship between the inertia force and the friction between the seed potato and the DLSPS is as follows:


(4) $$\{\begin{array}{c} f_{r}=\frac{m\times v_{1}^{2}}{\sqrt{(R_{1}+26{)}^{2}+{\left(\frac{T}{2}\right)}^{2}*10^{-3}}}\\ f_{f}=\mu \times f_{N}\\ f_{G}=m\times ɡ\\ \tan\beta =\frac{t/2}{R_{1}+26} \end{array}$$

where, *μ* is the friction coefficient of seed potato and DLSPS, and *μ* is taken as 0.445 (Qiu et al., 2023a); *R*_1_ is the radius of sprocket, mm; *t* is the thickness of seed potato, mm. Combining Equations 3 and 4 yields the following relationship (Equation 5):


(5) $$v=\sqrt{0.425cos\varphi -0.792sin\varphi }$$

As the angle (α) grows, the combined force applied by the DLSPS on the seed potato lessens, and at reaching a specified angle, the seed potato will detach and fall. An increase in the speed of the pickup line causes a matching rise in the inertia force at the original seed drop point. This causes the seed potato to be expelled from the DLSPS, which is designed to be positioned at a relatively small angle.

### 2.3 Operating algorithms

#### 2.3.1 Operating principle of the EMM

**Figure 7:**
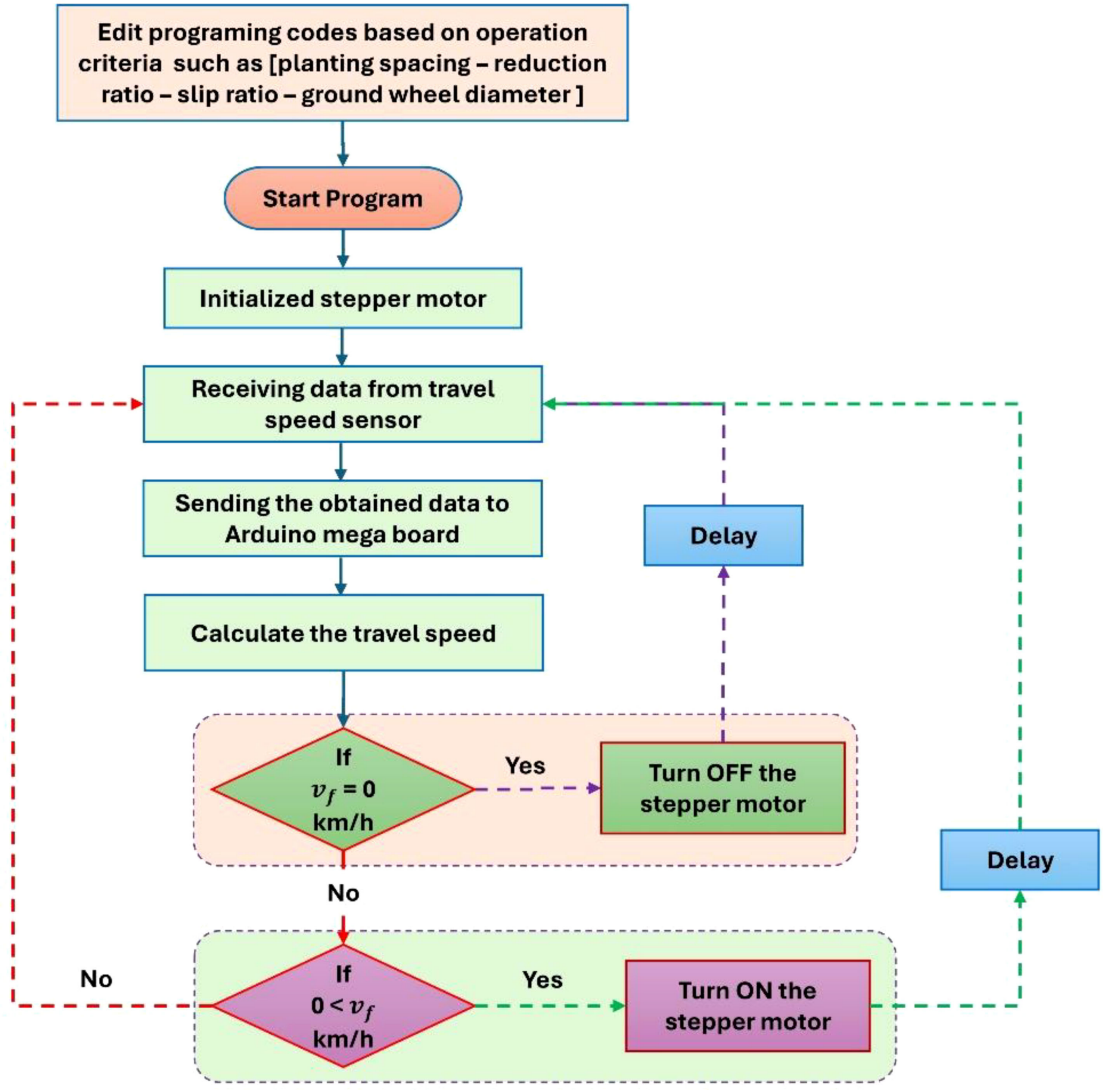
Operating algorithm of the MUPP.

**Figure 7** illustrates the open-loop control algorithm governing the MUPP, with the entire system being powered by an Arduino Mega 2560 microcontroller board. The control logic was developed using Arduino IDE 2.3.3, where the programming code was meticulously calibrated to account for critical operational parameters—including planting spacing, reduction ratio, slip ratio, and ground wheel diameter—before being uploaded to the microcontroller. The algorithm plays a pivotal role in real-time monitoring and control, processing sensor data to dynamically adjust the stepper motor speed in synchronization with the conveyor belt’s movement on the test bench. Additionally, the system incorporates a Wi-Fi-enabled data logging feature, transmitting real-time metrics such as planting speed and travel speed to a connected laptop for performance analysis and optimization. This integrated approach ensures precise, automated control while providing actionable insights for system refinement.

#### 2.3.2 Operating principle of the monitoring of the potato seed planting and replanting units

**Figure 8:**
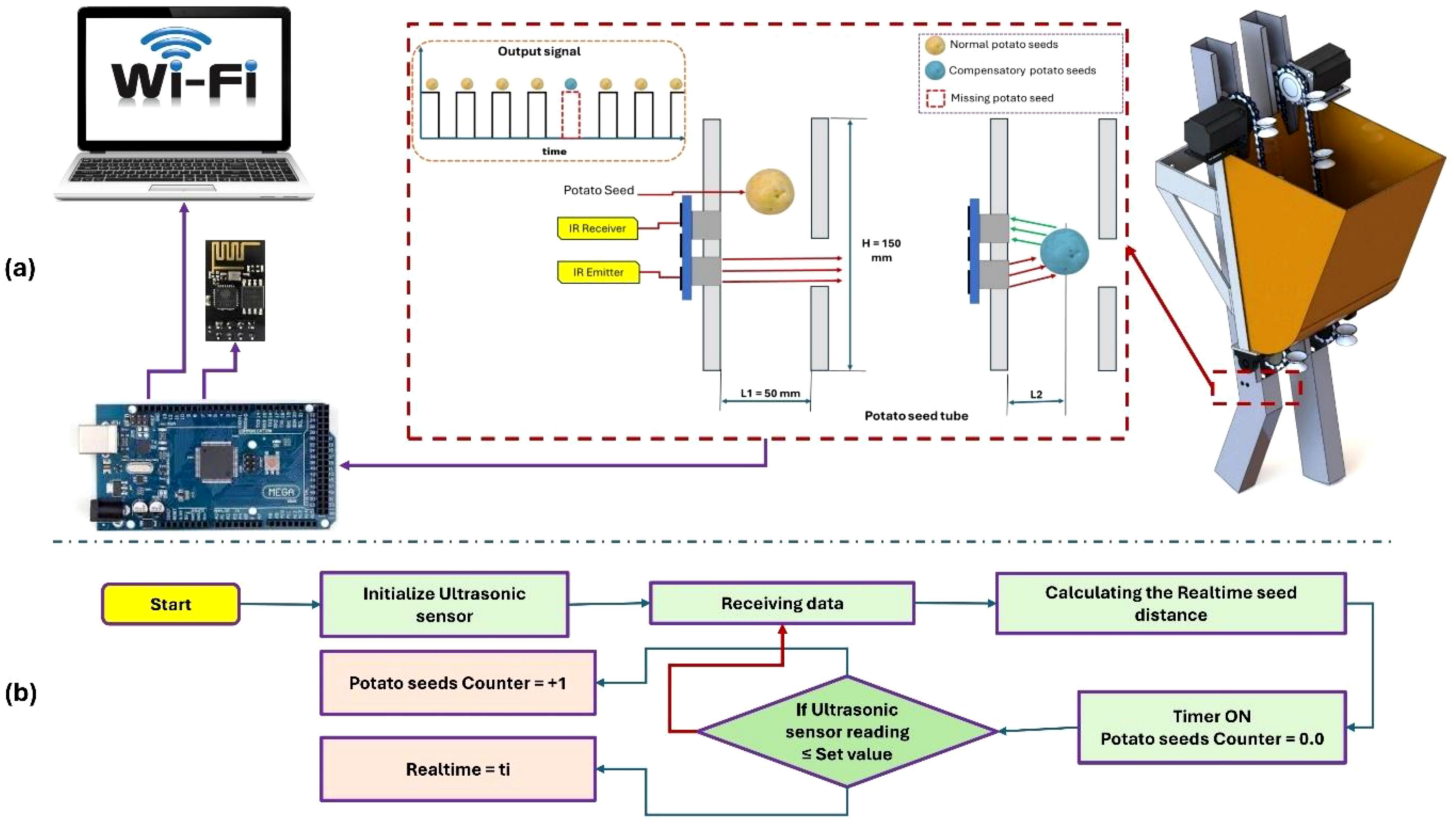
Operating algorithms of the monitoring of the potato seed planting and replanting units, **(a)**. Operating principle, and **(b)**. Operating algorithm.

**Figure 8** shows the operating algorithms for the monitoring of the potato seed planting and replanting units. The monitoring of potato seed metering such as qualified index (QI), mis-planting index (MPI), and replanting index (RI) was primarily conducted by assessing the quantity of input potato seeds, the rotational speed of the DLSPS via both the NPSGT and the CPSGT. A diffuse reflection ultrasonic sensor transformed this information into a pulse signal, enabling the system to tally the quantity of potato seeds and the frequency of DLSPS (theoretical planting number) placements in both NPSGT and CPSGT by observing the falling edge of the pulse signal. The time consumed was established by observing the rising and falling edges of a pair of pulses. The time for monitoring the planting sensor was documented as T_A_. The temporal interval of the seed guidance monitoring sensor was documented as T_B_. The interval time of the ultrasonic sensor T_A_ increased, leading to the omission of some seeding counts.

**Figure 9:**
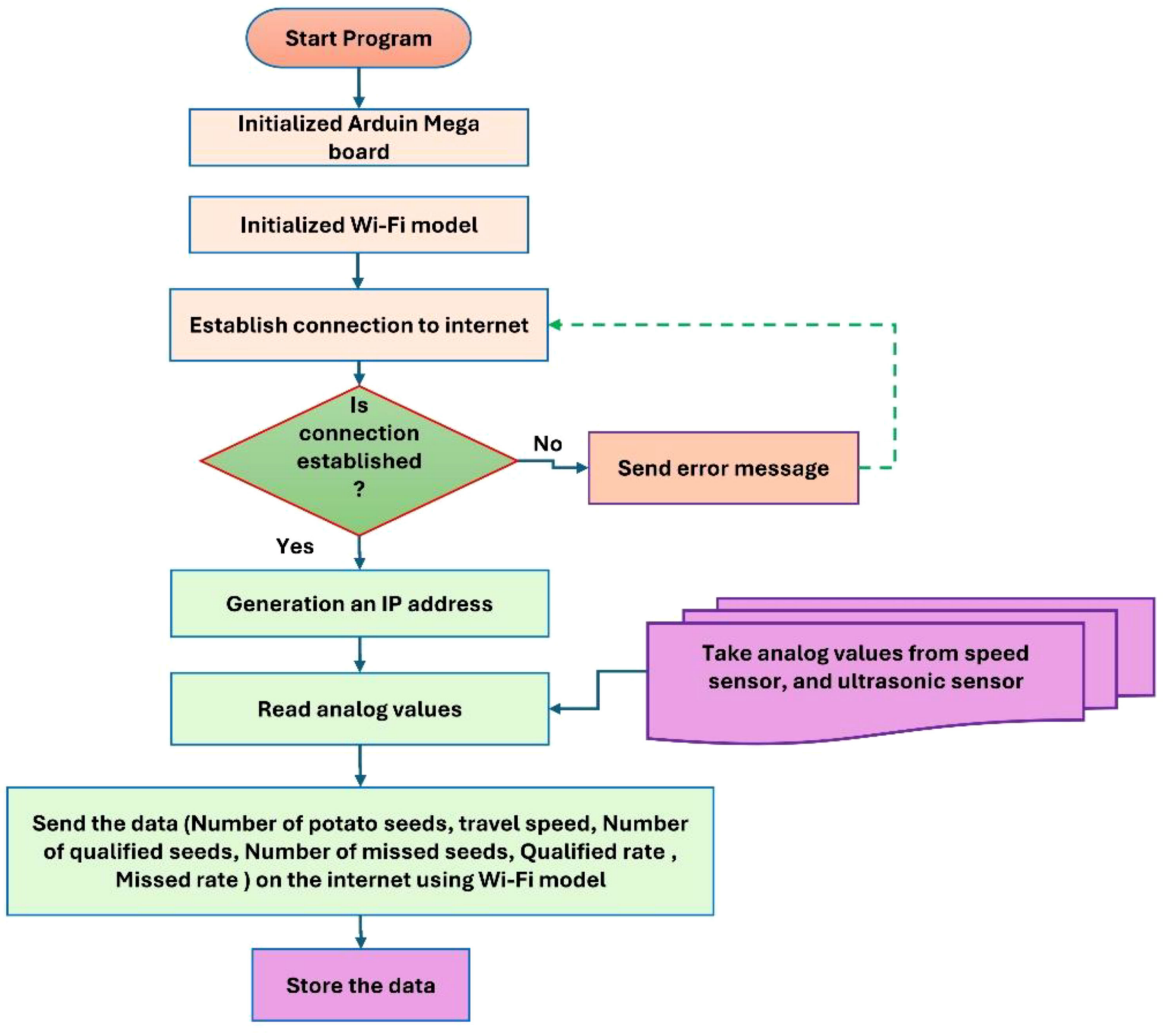
Operating algorithm for data transmission via Wi-Fi.

This project involved developing a display program in C++ using the free online software MIT App Inventor to provide real-time monitoring of planting parameters (QI, MPI, and RI). The software interface has four components related to the planting function: configuration of communication string numbers, establishment of seed parameters, oversight of operational parameters, and alarms for mis-planting. The communication string number configuration section facilitates the selection and activation or deactivation of the operational string number for equipment communication through Wi-Fi. The parameter configuration section is utilized to establish pertinent parameters, including the operational speed of stepper motors. Upon completion of the parameter design, it is stored in the operational memory, and the relevant parameters are accessed during program execution. The monitoring parameters encompass the quantity of potato seeds, the theoretical seed count, the planting interval, and the travel speed of the conveyor belt. The quantity of potato seeds is utilized to indicate the planting amount for each test in the seed metering apparatus. The theoretical quantity of potato seeds is employed to ascertain the number of seeds per test under regular operating conditions, ignoring mis-planting and the presence of multiple seeds. Upon mis-planting, the replanting device transmits a signal to activate the stepper motor associated with it, enabling the release of a single potato seed. **Figure 9** illustrates the operational algorithm for data transmission using Wi-Fi.

### 2.4 Laboratory experiment

**Figure 10:**
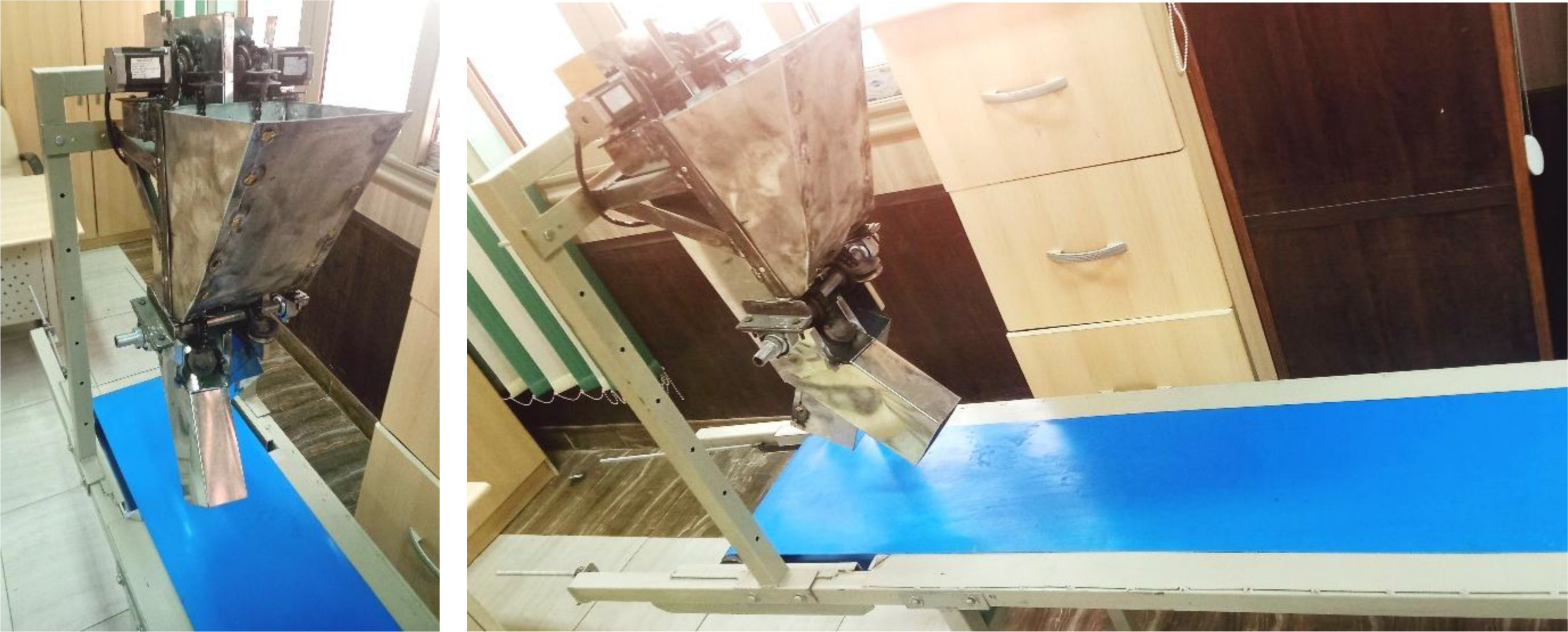
Laboratory evaluation of the developed EMM.

To assess the efficacy of the developed EMM, comparison experiments were executed on a constructed test bench (**Figure 10**), where the developed EMM is installed on a test bench of 4 meters in length, 50 cm in width, and 80 cm in height. The conveyor belt is 3.5 meters long and 30 cm wide, made of blue material. The conveyor functions with two rollers, each having a diameter of 12 cm and a length of 40 cm, featuring one ball bearing on either side. The conveyor belt operates using a 0.5 hp AC induction motor, with a dimmer controlling the operational speed per specifications. All laboratory evaluations done in the Department of Agricultural Engineering at Aswan University, Egypt, during October 2024. Using test bench method facilitates the efficient alteration of experimental levels and the effective regulation of non-experimental variables. Where the precision of the control system directly influences the potato seed planting operation. Numerous factors may influence the planting procedure in the field, including soil type, moisture content, crop residues, climatic conditions, and wheel-ground slide. The test bench was primarily conducted to assess the stability of the created control system, aiming to minimize any adverse effects on the planting process. The planting data and programming codes were sent to the controller. The data included planting spacing, reduction ratio, slip ratio, and ground wheel diameter. The experimental data can be sent to a laptop via USB cable and stored in Excel files or shown on the specified application using Wi-Fi.

**The laboratory tests include many tests such as:**

#### 2.4.1 Calibrating of the travel speed sensor and ultrasonic sensor

This examination entails calibrating and assessing the performance of the speed sensor at different travel speeds. Sensor calibration is a crucial procedure that must be conducted prior to operation. This technique is crucial to ensure the accuracy of the obtained results and to evaluate the effectiveness of the sensors in various operational conditions (Elwakeel et al., 2024b, a; Khater et al., 2024). In the current study, the speed sensor was calibrated at fourteen travel speeds ranging from 0.0 to 6.0 km/h, selected based on the optimal planting speed for the planting process. The speed of the conveyor belt was assessed by gauging the speed of the AC induction motor with a tachometer (model: UT372D, China). Additionally, the ultrasonic sensor was calibrated using a standard-length scale for distance measurement. During the calibration tests, various distances ranging from 0 to 30 centimeters were measured using both methodologies.

#### 2.4.2 Effect of travel speed and planting spacing in the QI, MPI, and RI

The aim is to determine the fluctuation in monitoring precision of the ultrasonic sensor for each potato seed flow at different travel speeds of 2.13, 3.07, 3.94, 5.09, and 6.11 km/h and planting spacing of 24.12, 31.06, 34.87, and 41.24 cm, to examine the accuracy enhancement and improvement trajectory of the ultrasonic sensor in conditions of high travel speed and narrow planting spacing. Twenty combinations were tested, each replicated a minimum of three times, utilizing 100 potato seeds per trial. The equations for computing each index were as follows, according to Tang et al. (2022), using Equations 6, 7, 8.


(6) $$Qualified\;index\;[Q,\;\%]=\frac{n_{0}}{N}\times 100$$

(7) $$Replanting\;index\;[M,\;\%]=\frac{n_{1}}{N}\times 100$$

(8) $$Mis-planting\;index\;[E,\;\%]=\frac{n_{2}}{N}\times 100$$

where, *n*_0_ is the potato seeds count in each test; *n*_1_ is the replanting potato seeds count in each test; *n*_2_ is the mis-planting potato seeds count in each test; and *N* is the total number of potato seeds in each test.

## 3 Results and discussion

### 3.1 Calibration of speed sensor and ultrasonic sensor

**Figure 11:**
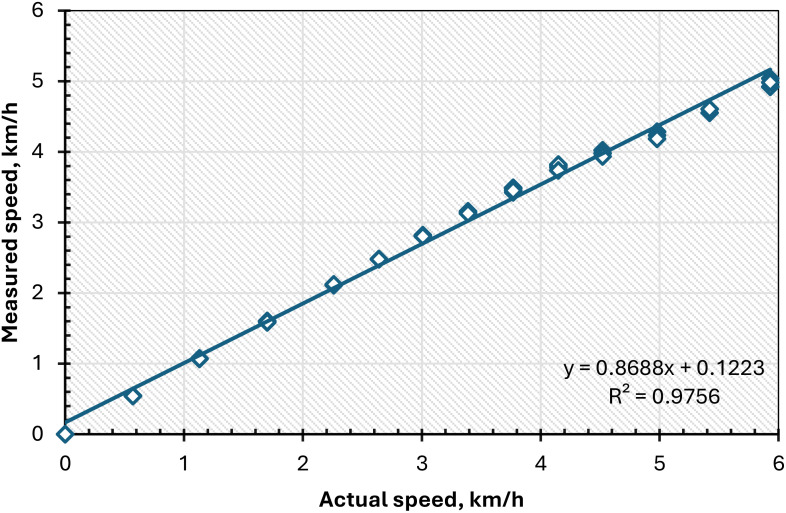
Calibration of the speed sensor against a high accurate commercial tachometer.

The infrared light speed sensor was utilized to measure the travel speed of the conveyor belt during the laboratory tests. This sensor has been utilized in numerous prior studies, including speed control for lane-switching mechanisms (Kalaipriyan et al., 2021), DC motor regulation via Arduino controllers (Ait Abdelkader and Rezzouk, 2023), and peed modulation of DC motors using pulse width modulation (Faroqi et al., 2018), Additionally, utilized in various agricultural machines, including the sugarcane transplanter machine (Elwakeel et al., 2023), and the multi-tasking agricultural robot (Kamatham et al., 2022). The speed sensor in the current study was calibrated at various operating speeds ranging from 0.0 to 6.0 km/h, selected based on the optimal planting speed for the planting process. The speed was assessed by gauging the speed of the AC induction motor with a tachometer (model: UT372D, China). As shown in **Figure 11**, the speed sensor’s measured speed shows a strong correlation with the actual speed recorded by the tachometer, reflected by a high R² value of 0.9756. At various travel speeds, the speed sensor demonstrated impressive performance, producing reliable linear regressions (y = 0.8688x) that closely matched the 1:1 line.

**Figure 12:**
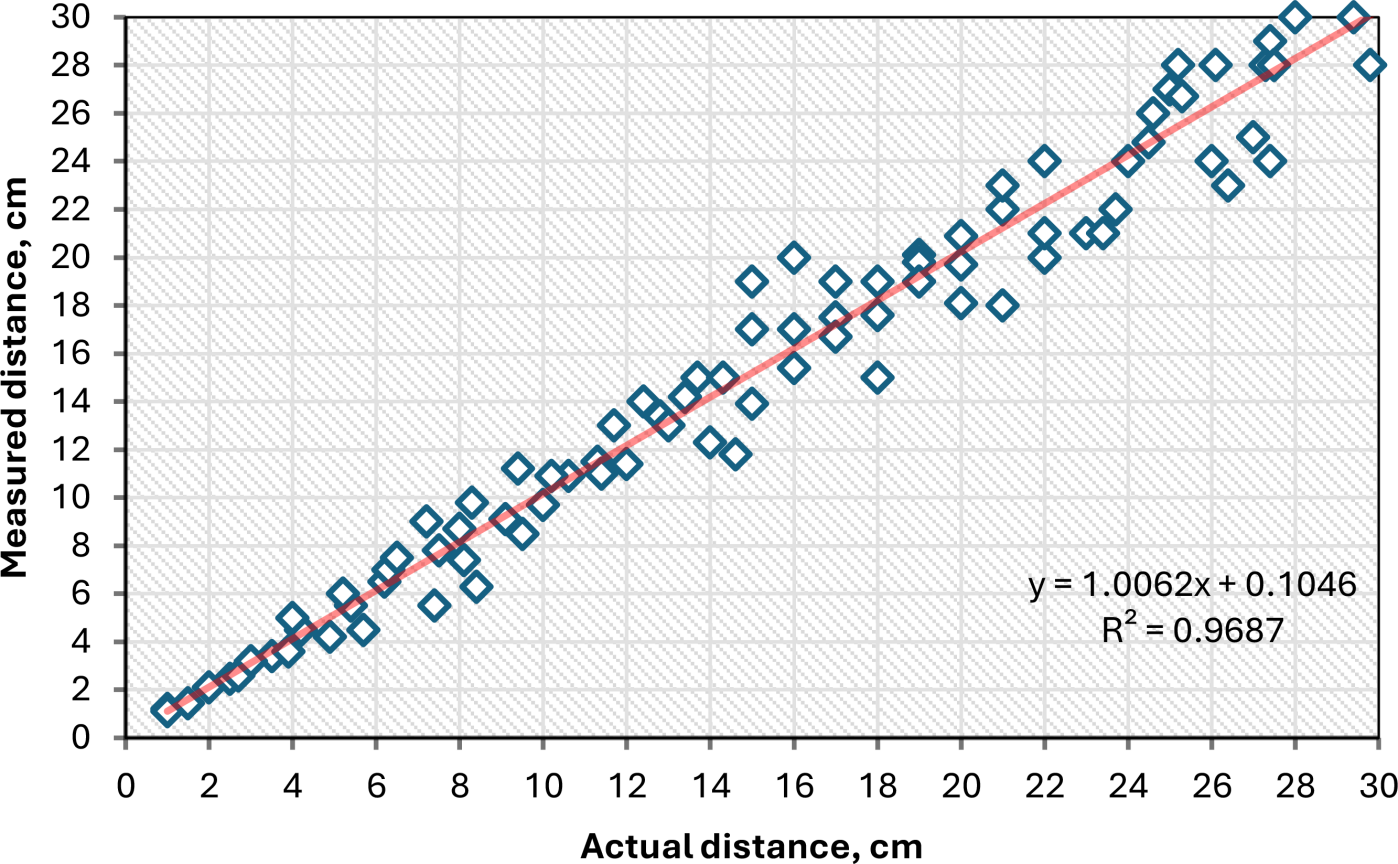
Calibration of the ultrasonic sensor against a length scale.

This study employed an ultrasonic sensor (model: HC-SR04) to precisely measure the quantity of potato seeds entering the seed guide tube. This method enabled the evaluation of qualified, missed, and replanted potato seeds. The selection of the ultrasonic sensor (model: HC-SR04) was predicated on its high accuracy and availability in local markets, where it is extensively utilized in diverse fields, including agricultural machinery (Elwakeel et al., 2024c; Yang et al., 2024), measuring distance (Hidayat et al., 2019; Kamal et al., 2019; Raza and Monnet, 2019), object detection (Hoomod and Al-Chalabi, 2017; Kulkarni et al., 2019; Raza and Monnet, 2019). The current study presents a comparison of the measured distance between the ultrasonic sensor and the traditional length scale, as illustrated in **Figure 12**. The regression equation shown in **Figure 12** is used to analyze and predict the relationship between the ultrasonic sensor readings and the traditional length scale. A steeper slope in the regression equation indicates a greater change in the measured distance. The R² value reflects the overall effectiveness of the regression equation, with a maximum possible value of 1. A higher R² indicates a better fit for the regression model. As illustrated in **Figure 12**, the ultrasonic sensor performed flawlessly across all tested distance values, demonstrating strong linear regressions (y = 1.0062x) that closely align with the 1:1 line. This is supported by a high R² value of 0.9687.

### 3.2 Effect of travel speed on QI, MPI, and RI

**Figure 13:**
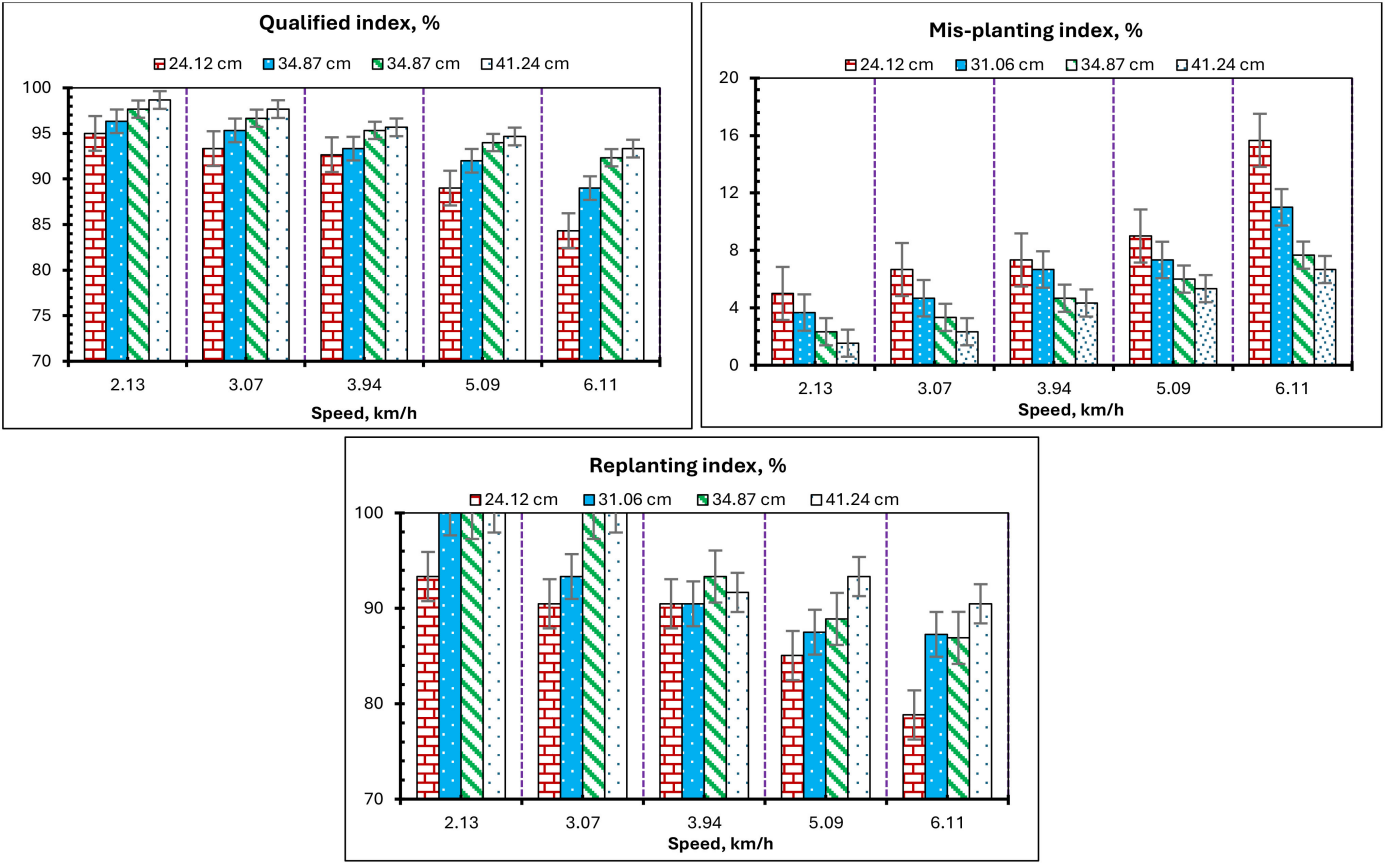
Effect of travel speed on the QI, MPI, and RI.

To explain the impact of the conveyor belt’s speed on the evaluation parameters (QI, MPI, and RI) during the laboratory assessment of the developed EMM, the evaluation parameters (QI, MPI, and RI) at different travel speeds are illustrated in **Figure 13**. In the laboratory tests, four planting spacings of 24.12, 31.06, 34.87, and 41.24 cm, and five conveyor belt travel speeds of 2.13, 3.07, 3.94, 5.09, and 6.11 km/h, were utilized experimentally on the test bench. According to the initial analysis of the test results: within the range of 2.13–3.07 km/h, the results are relatively stable, with travel speed exerting minimal influence on sensor monitoring accuracy; conversely, in the range of 3.94–5.09 km/h, the results exhibit significant variability, indicating that travel speed substantially affects the evaluation parameters (QI, MPI, and RI). Experimental results indicate that QI achieves the highest detection accuracy of 98.7% at a planting spacing of 41.24 cm and a travel speed of 2.13 km/h. The MPI exhibits great detection accuracy, ranging from 2.13 km/h to 3.07 km/h of the travel speed of the conveyor belt, with the minimum MPI recorded at 1.3% at a planting spacing of 41.24 cm and a travel speed of 2.13 km/h. The accuracy of mis-seed detection diminishes as the travel speed of the conveyor belt exceeds 3.07 km/h. Moreover, as illustrated in **Figure 13**, the RI is significantly influenced by a slight alteration when the travel speed is low. As the conveyor belt’s speed escalates, the RI will diminish. The analysis is performed due to an inverse association between travel speed and the rate of potato seed release per unit time (RI). At a travel speed of 2.13 km/h, the RI attained a maximum value of 100% at planting spacings of 31.06, 34.87, and 41.24 cm; additionally, the RI also reached a maximum value of 100% at planting spacings of 34.87, and 41.24 cm. At travel speeds of 5.09, and 6.11 km/h, the RI attained minimal values of 85% and 78.7%, respectively. Increasing travel speed while maintaining the same planting distance resulted in a higher quantity of potato seeds passing through the seed monitoring unit. Concurrently, the acceleration of potato seed movement led to a decline in evaluation parameters (QI, MPI, and RI), with this effect being particularly pronounced when the conveyor belt of the test bench operates at elevated speeds.

Comparable results were noted in other prior research, wherein Li et al. (2022b) indicated that when the operational speed for rice planting increased, the efficacy rate of the seed-metering device exhibited an upward trajectory, while the omission rate demonstrated a decrease trajectory. The primary reason is that increased sphericity of the seeds results in a shape more closely resembling a sphere, facilitating their entry into the metering system’s aperture. (Nan et al., 2016) indicate that the observed seed metering system achieves optimal seeding efficacy at a planter forward speed of 5.23 km/h. Wang et al. (2022) showed that when the pneumatic corn planter operates at speeds of 5 to 13 km/h, the average planting spacing qualification rate exceeds 95.81%, while the reseeding rate remains below 10.11%. Cay et al. (2018) and Cay et al. (2017) indicated that the seed plate speed is one of the most critical aspects affecting the uniformity of the planting spacing. Li et al. (2015); and Xie et al. (2021) indicated that the monitoring performance of the remote tracking system is superior at reduced speeds, while the monitoring error escalates with increased speed, thereby affecting its precision. Karimi et al. (2019) indicate that an increased speed of seeding will diminish the sensor’s accuracy in monitoring the process. Xie et al. (2021) indicate that an increase in seeding speed significantly alters the reliability of sensor monitoring. And Shuo et al. (2019) discovered that an increase in speed correlates with a decline in the qualified index and a rise in the mis-seed index.

### 3.3 Effect of planting spacing on QI, MPI, and RI

**Figure 14:**
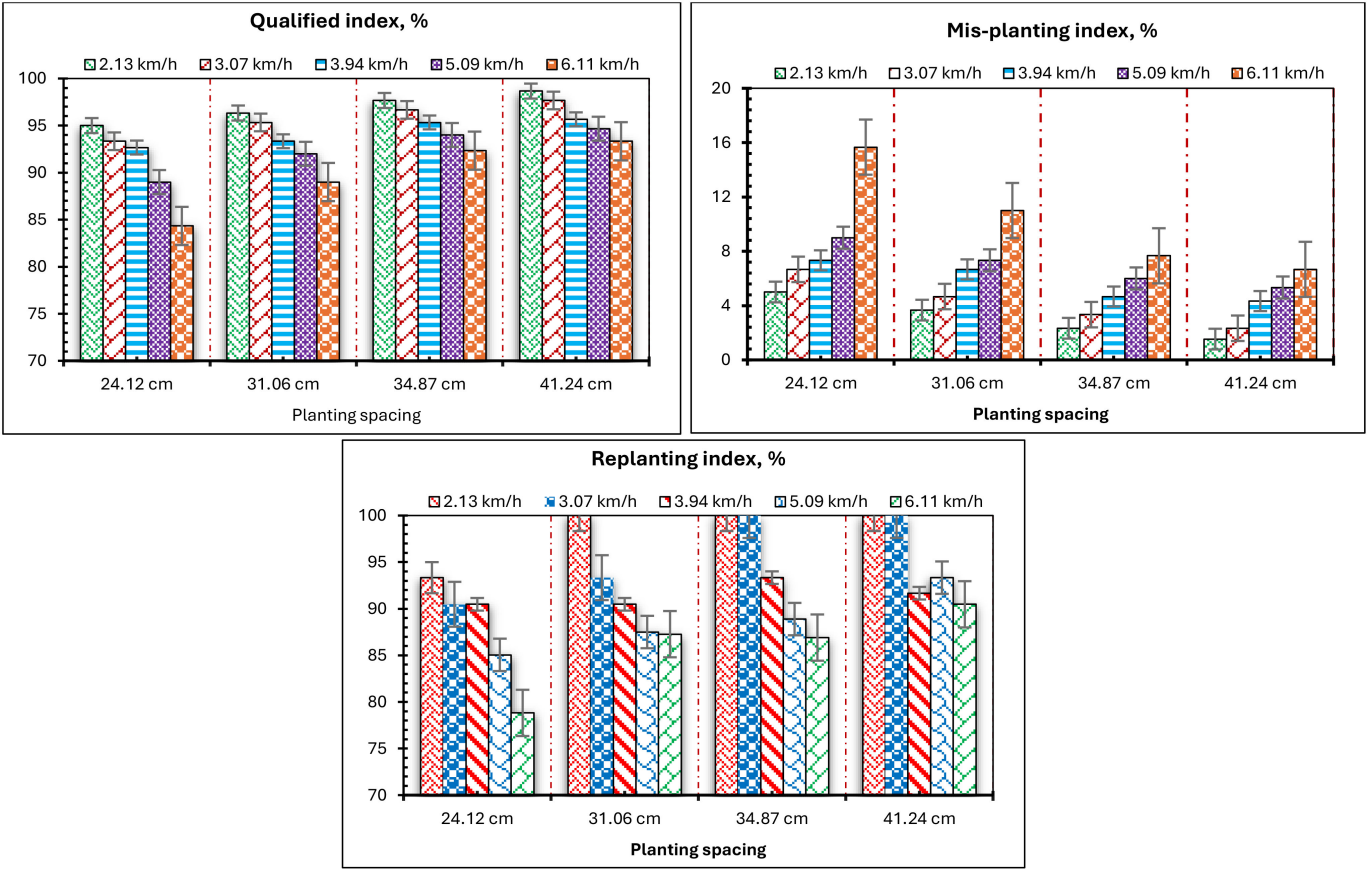
Effect of planting spacing on the QI, MPI, and RI.

To elucidate the impact of planting spacing on the laboratory assessment of the developed EMM regarding the evaluation parameters (QI, MPI, and RI), the evaluation parameters (QI, MPI, and RI) at different travel speeds are illustrated in **Figure 14**. In the laboratory tests, four planting spacings of 24.12, 31.06, 34.87, and 41.24 cm, and five conveyor belt travel speeds of 2.13, 3.07, 3.94, 5.09, and 6.11 km/h, were utilized on a test bench. According to the initial analysis of the results of bench tests: within the range of 31.06-34.87 cm, the results exhibit significant variability, with planting spacing exerting a pronounced influence on the evaluation parameters (QI, MPI, and RI); conversely, in the range of 24.12-31.06 cm, the results are comparatively stable, and seeding spacing has a minimal effect on the monitoring outcomes (QI, MPI, and RI). **Figure 14** illustrates that both QI and RI rose with greater planting space, although MPI exhibited an inverse trend. The maximum QI and RI were 98.7% and 100%, respectively, with a planting spacing of 41.24 cm and a travel speed of 2.13 km/h. Conversely, reducing the planting spacing from 24.12 cm to 41.24 cm at a travel speed of 2.13 km/h resulted in a decrease of approximately 3.7% in QI and 6.7% in RI. Moreover, reducing the planting spacing from 24.12 cm to 41.24 cm at a travel speed of 6.11 km/h resulted in a drop of approximately 9.6% in QI and 13.2% in RI. The trend of MPI diminished as planting spacing increased, as illustrated in **Figure 14**.

Comparable results were noted in numerous prior investigations; (Xie et al. (2021) indicated that at elevated speeds, variations in seeding spacing significantly influence the evaluation parameters (QI and MPI). The sensor’s monitoring accuracy will minimize when the seeding spacing reduces. (Xie et al., 2021) also found that when the seeding spacing is minimal, the reliability of sensor monitoring varies significantly. Subsequent analysis indicated that the frequency of seeds passing through the sensor is the primary determinant influencing sensor monitoring efficacy. The dependability of the sensor monitoring diminishes gradually as frequency escalates. Shuo et al. (2019) asserted that velocity significantly influences seeding performance, suggesting that an increase in speed results in greater dispersion of grain spacing, a reduction in the QI, and an elevation in the MPI.

### 3.4 Statistical analysis

A two-factor analysis of variance was conducted on travel speed and planting spacing based on the preliminary data above. Additionally, investigate the evolving regulations on the impact of travel speed and planting spacing on the evaluation parameters (QI, MPI, and RI). Utilize the statistical analysis program IBM SPSS 16.0 to evaluate the acquired data and employ Duncan’s multiple comparison analysis methods for data processing. The study employed a factorial design with two factors: planting spacing (4 levels) and travel speed (5 levels), resulting in 20 treatment combinations. Each combination was replicated 3 times, totaling 60 experimental units. This replication ensured sufficient power for detecting significant effects. The analysis of variance (ANOVA) was conducted to evaluate the effects of planting spacing, travel speed, and their interaction on the measured response variables. Key findings are summarized below (main Effects and Interactions):

➢ Planting Spacing: Highly significant effect (F=197.042, p < 0.001) in the first analysis, with similar significance in subsequent models.
➢ Travel Speed: Also, highly significant (F = 240.250, p < 0.001) in the first analysis, though significance varied across models (e.g., F=3.509*F*=3.509, p=0.015*p*=0.015 in the third analysis).
➢ Interaction (Spacing × Speed): Significant in the first two analyses (p<0.001*p*<0.001) but non-significant in the third (p=0.999*p*=0.999), suggesting context-dependent effects.

1. Model Fit: The corrected model explained a significant proportion of variance in all analyses (p < 0.001), though the adjusted R^2^ was low (0.042) in the third analysis, indicating potential unaccounted variability.

2. Error Terms: Error degrees of freedom (df = 40) and mean squares were consistent with the replicated design.

**Table 1** demonstrates that travel speed, planting spacing, and their interaction have highly significant impacts on the evaluation parameters (QI and MPI) (p < 0.001), leading to the acceptance of the null hypothesis, which posits that these factors significantly influence QI and MPI. The travel speed and planting spacing were shown to considerably influence the QI and MPI of the designed EMM. Additionally, the travel speed significantly affects the RI (p < 0.05); however, the interaction between travel speed and planting spacing does not significantly impact the RI.

**Table 1:** ANOVA results in the effect of travel speed and planting spacing on the QI, MPI, and RI.

Evaluation parameters	S.O.V.	Sum of Squares	df	Mean Square	F	Sig.
**QI**	Intercept	528094.017 ^a^	1	528094.017	1320235.042	0.000 **
	Corrected model	664.983	19	34.999	87.498	0.000 **
	**Planting spacing**	236.450	3	78.817	197.042	0.000 **
	**Travel speed**	384.40	4	96.100	240.250	0.000 **
	Spacing * Speed	44.133	12	3.678	9.194	0.000 **
	Error	16.0	40	0.400		
	Total	528775.0	60			
	Corrected total	680.983	59			
**MPI**	Intercept	606.637 ^a^	19	31.928	56.745	0.000**
	Corrected model	197.741	3	65.914	117.145	0.000**
	**Planting spacing**	2203.416	1	2203.416	3916.024	0.000**
	**Travel speed**	362.731	4	90.683	161.166	0.000**
	Spacing * Speed	46.165	12	3.847	6.837	0.000**
	Error	22.507	40	0.563		
	Total	2832.560	60			
	Corrected total	629.144	59			
**RI**	Intercept	1915.917^a^	19	100.838	1.137	0.355
	Corrected model	486.450	3	162.150	1.829	0.157
	**Planting spacing**	508760.417	1	508760.417	5737.899	0.000**
	**Travel speed**	1244.500	4	311.125	3.509	0.015
	Spacing * Speed	184.967	12	15.414	0.174	0.999
	Error	3546.667	40	88.667		
	Total	514223.000	60			
	Corrected total	5462.583	59			

a R Squared = 0.351 (Adjusted R Squared = 0.042).

** highly significant at P ≤ 0.001. (The color shading and bold values highlight the significance of travel speed, planting distances, and their interaction).

### 3.5 Estimated cost of the developed EMM

As shown in **Table 2**, the total cost of the developed EMM was estimated based on local conditions in Egypt. Where the main operating and control system consists of seven parts, including an Arduino board, stepper motor, stepper motor driver, speed sensor, ultrasonic sensor, Bluetooth unit, and power supply. The total cost of this system was approximately $130 USD. The cost of the measuring system is much lower than the cost of the laptop that is used for the same purposes if we used machine learning. This affordability makes the developed EMM an attractive option for local applications, particularly in agricultural sectors where budget constraints are common. Furthermore, the ease of integration with existing technologies enhances its practicality for users seeking efficient monitoring solutions. This efficiency not only supports farmers in optimizing their resources but also promotes sustainable practices that can lead to improved crop yields. Ultimately, the EMM system’s combination of cost-effectiveness and functionality positions it as a valuable tool in advancing agricultural productivity.

**Table 2:** Estimated cost of the developed EMM.

No.	Quantity	Component	Model	Cost, USD
1.	1	Arduino board	Arduino Mega 2560	35
2.	2	Stepper motor	Nema 23	50
3.	2	Stepper motor driver	TB6600	20
4.	1	Speed sensor	LM393IC	5
5.	2	Ultrasonic sensor	HC-SR04	10
6.	1	Bluetooth unit	HC-05	5
7.	1	Power supply	………….	5
Total estimated costs				130

## 4 Conclusion

This study developed and evaluated an optimized, high-precision variable-rate electronic metering mechanism (EMM) for potato replanting, capable of detecting and correcting mis-planted seeds through three performance indices: qualified planting (QI), mis-planting (MPI), and replanting (RI). Where the developed EMM was evaluating using a test bench at four planting spacings of 24.12, 31.06, 34.87, and 41.24 cm, and five conveyor belt travel speeds of 2.13, 3.07, 3.94, 5.09, and 6.11 km/h. A dedicated electronic monitoring and counting circuit was developed to evaluate system performance through three key metrics: qualified planting index (QI, successful seed placement), mis-planting index (MPI, failed placements), and replanting index (RI, successful corrections of MPI)According to the obtained results, within the range of 2.13–3.07 km/h, the results are relatively stable, with travel speed exerting minimal influence on sensor monitoring accuracy; conversely, in the range of 3.94–5.09 km/h. The experimental results show that we achieved the highest QI, PI, and the lowest MPI at a planting spacing of 41.24 cm and a travel speed of 2.13 km/h. At a planting spacing of 41.24 cm and a travel speed of 2.13 km/h, the maximum QI and RI reached 98.7% and 100%, respectively. Conversely, reducing the planting spacing from 24.12 cm to 41.24 cm at a travel speed of 2.13 km/h resulted in a decrease of approximately 3.7% in QI and 6.7% in RI. Moreover, reducing the planting spacing from 24.12 cm to 41.24 cm at a travel speed of 6.11 km/h resulted in a drop of approximately 9.6% in QI and 13.2% in RI. Furthermore, the total cost of the developed system was approximately $130 USD. Finally, it can be concluded that increasing the travel speed of the conveyor belt and decreasing the planting spacing led to a decline in evaluation parameters (QI, MPI, and RI).

## Data Availability

The original contributions presented in the study are included in the article/supplementary material. Further inquiries can be directed to the corresponding authors.
